# Preventive effects of compound probiotics supplementation on migraine: Mendelian randomization analysis and experimental evidence

**DOI:** 10.3389/fmicb.2026.1939532

**Published:** 2026-10-21

**Authors:** Lu Bai, Qingteng Zhang, Jingyuan Zhu, Wenqiang Zhang, Zhenwu Cui, Tian Jia, Chaokun Li, Zhiyue Guo

**Affiliations:** 1 Henan Medical University, Xinxiang, China; 2 Department of Pathology, The Fifth Affiliated Hospital of Anhui Medical University, Fuyang, China; 3 The Third Clinical College, Henan Medical University, Xinxiang, China; 4 The First Affiliated Hospital of Zhengzhou University, Zhengzhou, China; 5 Department of Physiology and Pathophysiology, School of Basic Medical Sciences, Henan Medical University, Xinxiang, China

**Keywords:** brain structure, gut microbiota, Mendelian randomization, migraine, probiotic

## Abstract

**Introduction:**

Migraine imposes a substantial economic and societal burden. The microbiota-gut-brain axis (MGBA) has emerged as a key potential mechanism, yet its causal relationships with migraine and the underlying pathways remain unclear.

**Methods:**

We performed Mendelian randomization (MR) to assess the causal effects of gut microbiota on migraine, and further conducted mediation analysis to identify key mediating brain regions. In addition, we performed *in vivo* mouse experiments, in which we induced migraine-like pain via nitroglycerin (NTG) and investigated changes in gut microbiota, mechanical pain threshold, and regional brain activation following pretreatment with composite probiotics.

**Results:**

MR analysis identified eight gut microbial taxa causally associated with migraine and its subtypes. Mediation analysis suggested that the association between genetically predicted *Paraprevotella* abundance and migraine risk was partially mediated by ICVF in the posterior insular cortex. In NTG-induced migraine mice, composite probiotics ameliorated gut dysbiosis and maintained *Paraprevotella* abundance, while elevating mechanical pain thresholds in both periorbital and hind paw region. Probiotics intervention also suppressed c-Fos expression in the pIC, Sp5c, PVT, and mPFC. Additionally, it specifically decreased CGRP immunoreactivity in the Sp5c.

**Conclusion:**

This study provides genetic and experimental evidence supporting a potential role of the gut microbiome in migraine and the potential of microbiome-based strategies for migraine prevention.

## 1 Introduction

Migraine is a primary headache disorder characterized by moderate to severe recurrent headaches. The condition is classified into two primary clinical subtypes, migraine with aura (MA) and migraine without aura (MO). MA is frequently preceded by transient neurological symptoms such as visual or sensory disturbances (Lucas, 2021). In contrast, MO lacks aura but is more common (Paemeleire et al., 2023). These attacks are often accompanied by photophobia, phonophobia, nausea, and emesis (Dodick, 2018). Recent advances in microbiota-gut-brain axis (MGBA) research indicate that alterations in intestinal flora may participate in the onset and progression of migraine, but the underlying mechanisms remain unclear.

Growing evidence points to a role of dynamic gut microbiota changes in altering brain physiology, and this influence has also been linked to the pathophysiology of migraine (Zhu et al., 2020). Currently, an observational study has established an association between migraine and gut microbiota dysbiosis (Faraji et al., 2012). Furthermore, several studies have linked migraine to various gastrointestinal disorders such as *helicobacter pylori* infection (Wang et al., 2024), irritable bowel syndrome (IBS) (Arzani et al., 2020), and celiac disease (Qasim et al., 2022), a connection that may involve the MGBA.

For most of the 20th century, migraine was widely conceptualized as a vascular disorder characterized by cranial arterial dilation, yet growing evidence contradicts this hypothesis (Goadsby, 2009). Many neuroimaging studies have revealed that patients with migraine exhibit distinct structural alterations (Rahimi et al., 2022; Zhang et al., 2023; Christensen et al., 2024; Xiong et al., 2025). Nevertheless, the exact mechanisms underlying these morphological abnormalities remain poorly elucidated. A growing body of evidence indicates that gut microbiota plays a critical role in modulating brain structure. For instance, gut dysbiosis has been linked to cortical atrophy and disruptions in functional connectivity in schizophrenia (Li et al., 2021). Similarly, in the aging population, the depletion of specific bacterial genera has been linked to hippocampal atrophy and white matter deterioration, implying that microbial imbalances may contribute to the structural underpinnings of cognitive decline (Liang et al., 2022). However, the link between gut microbiota, brain structure, and migraine susceptibility remains unestablished. Considering that structural brain lesions are frequently coupled with altered neural activity, we combined brain structural analysis and c-Fos activity assessment to elucidate the neural correlates of migraine.

Traditionally, the randomized controlled trial (RCT) has been regarded as the gold standard for establishing causal inference. Yet, practical and ethical constraints often render RCTs difficult to conduct. Mendelian randomization (MR) is an epidemiological method that leverages genetic variants as instrumental variables (IVs) for causal inference (Sanderson et al., 2022). It estimates the causal effect of an exposure on an outcome by utilizing genetic IVs, typically single nucleotide polymorphisms (SNPs) that are robustly associated with the exposure (Smith and Ebrahim, 2003). A key strength of this method is minimizing confounding and reverse causation, since alleles assort randomly at conception and are independent of environmental factors.

In this study, we first employed a two-sample MR analysis to assess the causal relationship between gut microbiota and migraine/MA/MO. Subsequently, we performed two-step MR and mediation analyses, which identified potential brain structural traits that may mediate the genetically predicted association between gut microbiota and migraine. Finally, in the NTG-induced migraine mouse model, prophylactic supplementation with compound probiotics reduced mechanical pain sensitivity and suppressed neuronal activation in distinct brain regions. These findings offer a theoretical foundation for considering probiotics as a preventive intervention for migraine.

## 2 Materials and methods

### 2.1 Study overview

Figure 1 provides a summary of the study’s design. To investigate the genetic causality between exposures and outcomes, we performed two-sample MR analyses leveraging publicly accessible genome-wide association studies (GWAS) summary statistics, with full details of all datasets provided in Supplementary Table 1. Our analysis proceeded in several steps. First, we examined the causal effect of the gut microbiome on migraine and then assessed brain structure phenotypes. These analyses were replicated in subtype-specific analyses for MA and MO. Finally, we performed MR mediation analysis to investigate which brain structural phenotype alterations mediate the causal pathway from the gut microbiome to migraine. To provide complementary experimental evidence, we used 8-week-old male C57BL/6J mice for *in vivo* tests. Mice received preventive compound probiotics pretreatment, followed by repeated nitroglycerin (NTG) injections to induce migraine-like pain. We then assessed mechanical pain thresholds, gut microbiome composition, and brain region-specific neuronal activation.

**Figure 1:**
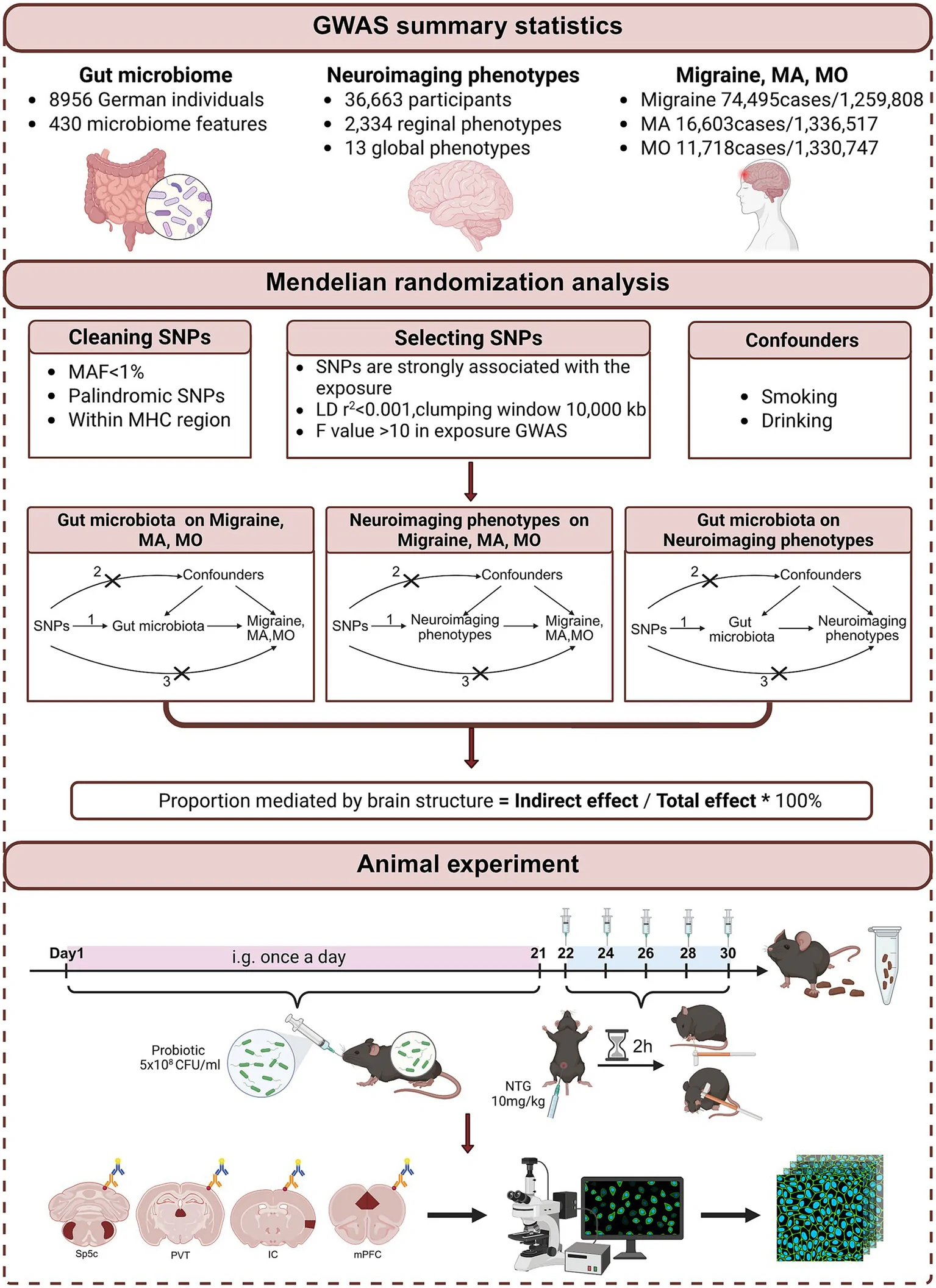
Overview of the study design. 1: the IVs must be strongly associated with the exposure; 2: the IVs must be independent of the potential confounders of the association between the exposure and outcome; 3: the IVs should not be associated with the outcome directly. Created with BioRender.com. MAF, minor allele frequency; MHC, major histocompatibility complex; LD, linkage disequilibrium.

### 2.2 Data sources

Genetic instruments for the gut microbiota were derived from a GWAS conducted by Rühlemann et al., which involved 8,956 individuals from a German population. This study performed a comprehensive GWAS of 430 microbial features (Supplementary Table 2). These features were defined using a multi-level approach, encompassing taxonomy from phylum to genus, as well as finer resolutions achieved via sequence similarity clustering (97% and 99%). Full summary statistics for these 430 microbial features are publicly accessible at https://www.ebi.ac.uk/gwas/publications/33462482 (Rühlemann et al., 2021).

The GWAS summary statistics for migraine were sourced from the largest available dataset to date, which included clinically defined migraine, MA, and MO. This comprehensive study integrated data from multiple international cohorts and encompassed 1.3 million individuals, including 74,495 overall migraine cases, of which 16,603 were classified as MA and 11,718 as MO (Bjornsdottir et al., 2023).

The GWAS summary statistics for brain structure were obtained from a large-scale meta-analysis, which encompassed 13 global and regional cortical MRI phenotypes. These phenotypes were assessed across 180 bilaterally averaged brain regions in a total of 36,663 individuals from the UK Biobank and Adolescent Brain Cognitive Development (ABCD) studies. The phenotypes comprise eight macrostructural measures: cortical thickness (CT), surface area (SA), cortical volume (Vol), folding index (FI), intrinsic curvature index (ICI), local gyrification index (LGI), mean curvature (MC), and Gaussian curvature (GC). They also include five microstructural measures: fractional anisotropy (FA), mean diffusivity (MD), isotropic volume fraction (ISOVF), intracellular volume fraction (ICVF), and orientation dispersion index (ODI) (Warrier et al., 2023). Further details are provided in Supplementary Table 3.

### 2.3 Selection and validation of instrumental variables

We removed variants with minor allele frequency (MAF) < 1% from the GWAS summary statistics. Subsequently, to circumvent ambiguous allele harmonization resulting from strand orientation issues, we excluded palindromic SNPs possessing MAF values close to 50% (Hartwig et al., 2016). We also removed the SNPs in the major histocompatibility complex (MHC) region (chromosome 6: 24–35 Mb).

For valid causal inference, MR requires genetic instruments to satisfy three core assumptions: (1) strong association with the exposure; (2) independence from potential confounders; (3) no direct pleiotropic effects (Zheng et al., 2017). Accordingly, we preprocessed IVs as follows. First, genetic instruments for gut microbiota were selected using a significance threshold of 1 × 10^−5^, whereas a more rigorous threshold of 5 × 10^−8^ was applied for brain structure-related variants. Second, we performed linkage disequilibrium (LD) pruning (r^2^ < 0.001, 10,000 kb window) using the TwoSampleMR package with European LD reference from 1,000 Genomes. Third, SNPs demonstrating associations with confounding factors were removed from the analysis. Previous studies have shown that smoking and drinking may affect migraine (Vives-Mestres et al., 2022; Yuan et al., 2022; Wu et al., 2023). The confounder associated SNPs were acquired from the MRC IEU OpenGWAS database (Elsworth et al., 2020), accessible at https://gwas.mrcieu.ac.uk/, with full details provided in Supplementary Table 4.

We additionally employed the F-statistic to evaluate the strength of the IVs. The F-statistic value exceeding 10 indicates a low risk of weak instruments. The F-statistic was calculated as (Guo et al., 2022):

$F=\frac{R^{2}}{1-R^{2}}\times \frac{n-k-1}{k}$ , $R^{2}=\frac{\beta^{2}}{\beta^{2}+se^{2}\times n.}$

In this formula, R^2^ quantifies the cumulative explanatory power of all genetic instruments on the exposure, calculated as the sum of individual variant R^2^ values. Each variant’s contribution is computed using its effect size (β), sample size (*n*) and standard error (se) from the exposure GWAS, while k denotes the total number of instruments included.

### 2.4 MR analysis

We performed MR analyses using the ‘TwoSampleMR’ R package (Guo et al., 2022). The Wald ratio was used for single-SNP instrument (Wald, 1940). For exposures with two or three IVs, the fixed-effect IVW method was employed as the primary method for causal inference (Borenstein et al., 2010). When the number of IVs was more than three, we relied on the IVW method with a random effect model (Burgess et al., 2013). Since the IVW estimate may be biased in the presence of horizontal pleiotropy, we enhanced the robustness of the causal inference by implementing three additional MR methods. The weighted median method provides consistent causal estimates even when up to 50% of the genetic instruments are invalid (Bowden et al., 2016). The weighted mode method demonstrates superior performance in maintaining consistent estimates under the relaxed IV assumption, characterized by reduced bias and enhanced control over type I error rates (Hartwig et al., 2017). The MR-Egger approach derives causal estimates from the slope of its regression line, maintaining robustness even when all genetic instruments potentially exhibit pleiotropic effects (Burgess and Thompson, 2017). A causal relationship was inferred if estimates from the different MR methods were consistent in direction and the IVW *p*-value passed the Bonferroni correction, ensuring the robustness of the findings.

### 2.5 Mediation analysis

We performed mediation analysis to quantify the pathway from the gut microbiome to migraine via brain structure. We first quantified the causal effect of the gut microbiome on the MRI phenotype (β₁) and then the effect of the MRI phenotype on migraine (β₂). The indirect effect mediated through the MRI phenotype was calculated as the product of coefficients (β₁ × β₂) (VanderWeele, 2016). We determined the mediated percentage by dividing the indirect effect (β₁ × β₂) by the total effect (β₃). The standard errors and 95% confidence intervals for the indirect effect and proportion mediated were estimated using the delta method (Carter et al., 2019).

### 2.6 Multivariable MR analysis

For the variables involved in the mediation analysis, multivariable MR (MVMR) was further performed to estimate their mutually adjusted direct effects on the outcome. Conditional F statistics were calculated to assess instrument strength. Analyses were conducted using the MVMR package in R. In MVMR analysis, we considered results with a *p* value of <0.05 as significant.

### 2.7 Sensitivity analysis

We performed a sequence of sensitivity analyses to verify the robustness of the MR results. Specifically, MR-Egger intercept test and MR-PRESSO global test were used to examine horizontal pleiotropy, with a significance level of *p* < 0.05 (Bowden et al., 2015; Verbanck et al., 2018). Additionally, we employed Cochran’s Q statistic to test heterogeneity in the SNP-specific effects for both the IVW and MR-Egger analyses (Kulinskaya and Dollinger, 2015). Potential influential variants were further evaluated using outlier-detection procedures, including RadialMR and MR-PRESSO where applicable. SNPs identified as outliers were removed, and the MR and sensitivity analyses were repeated. Results before and after outlier removal were both reported. Since participants from the UK Biobank may have contributed to both the brain imaging GWAS and the migraine GWAS, potential sample overlap could not be excluded. MRlap was therefore performed as an additional sensitivity analysis for associations between brain structure and migraine (Mounier and Kutalik, 2023). Within the MRlap framework, the observed and corrected estimates were compared, and the difference between them was tested. Steiger directionality analysis was performed for the three key associations involved in the mediation pathway (Hemani et al., 2017). Reverse MR analyses were additionally performed to assess potential reverse causality using the same analytical procedures as the forward MR analyses.

### 2.8 Chronic migraine models

We ordered male C57BL/6J mice (8 weeks old) from SPF Biotechnology Co. Ltd. (Beijing, China). All mice were maintained under specific pathogen-free conditions with a 12 h light/dark cycle and had free access to food and water. The experimental procedures were reviewed and approved by the Animal Ethics Committee of Henan Medical University (No. NYYLL-20260456, 3 April 2026). Referring to previously reported methods, we established a migraine mouse model by intermittent intraperitoneal (i.p.) injection of NTG (Pan et al., 2022). The stock solution (5 mg/mL in 30% alcohol, 30% propylene glycol, and distilled water, Beijing Yimin, China) was diluted with saline to 1 mg/mL for injection. NTG was administered i.p. at 10 mg/kg every other day for a total of five doses.

### 2.9 *In vivo* composite probiotics administration

The composite probiotics (Shanxi Yanhexiang, China) contained *Lactobacillus acidophilus*, *Lactobacillus casei*, *Lactobacillus rhamnosus*, *Bifidobacterium lactis*, *Bifidobacterium adolescentis* and *Bifidobacterium longum*. According to a previous study, the powder was dissolved in double distilled water (ddH_2_O) to obtain a bacterial suspension to a final concentration of 5 × 10^8^ CFU/mL (Zheng et al., 2024). Mice in probiotics intervention group were administered the prepared suspension via oral gavage for 21 consecutive days.

### 2.10 Microbial 16S rRNA sequencing

Fecal samples were collected under aseptic conditions and immediately stored at −80 °C until further processing. Genomic DNA was extracted using the QIAamp Fast DNA Stool Mini Kit (Qiagen, Germany) following manufacturer’s standardized protocols. The hypervariable V3-V4 regions of bacterial 16S rRNA genes were amplified via PCR using barcoded primers 338F (5′-ACTCCTACGGGAGGCAGCA-3′) and 806R (5′-GGACTACHVGGGTWTCTAAT-3′) with FastPfu DNA polymerase. The resulting amplicons were then sequenced on an Illumina NovaSeq 6,000 platform. Raw reads with >10% ambiguous bases or >40% bases with Q ≤ 20 were removed. The qualified paired-end clean reads were further filtered and assembled using FLASH (v1.2.11) to generate high-quality clean tags. All valid tags were clustered into operational taxonomic units (OTUs) at a similarity threshold of 97% using UPARSE (v9.2.64). The most abundant sequence of each OTU was selected as the representative sequence, which was taxonomically annotated using the RDP Classifier against the SILVA database (v2.2) with a confidence threshold set at 0.8 to 1.0. Alpha diversity indices were calculated using the MicrobiotaProcess package (v1.18.0) in R software (v4.4.2). Beta diversity was evaluated using Bray–Curtis dissimilarity and visualized by PCoA. Group differences were assessed by PERMANOVA, and homogeneity of multivariate dispersion was evaluated by PERMDISP. Differential microbial abundance was analyzed using linear discriminant analysis effect size (LEfSe), with the linear discriminant analysis (LDA) score set at a threshold of ≥ 2.0.

### 2.11 Quantitative PCR analysis

Genomic DNA was extracted from fecal samples using the TIANamp Stool DNA Kit (DP328; TIANGEN Biotech, Beijing, China) according to the manufacturer’s instructions. The relative abundance of *Paraprevotella* was determined by SYBR Green-based quantitative PCR (qPCR) using taxon-specific primers targeting the bacterial 16S rRNA gene, with universal bacterial 16S rRNA gene amplification used for normalization. The primer sequences were as follows: *Paraprevotella*, forward 5′-AGGGGCAGCATGGACCC-3′ and reverse 5′-CCTTTCAGGAGACTATCCCGGA-3′; total bacteria 16S, forward 5′-GGTGAATACGTTCCCGG-3′ and reverse 5′-TACGGCTACCTTGTTACGACTT-3′. The qPCR cycling conditions consisted of an initial denaturation step of 95 °C for 3 min followed by 40 cycles of 95 °C denaturation for 10 s and an annealing/extension step of 30 s at 60 °C. Technical replicates were averaged for each sample, and the relative abundance of *Paraprevotella* was calculated as $2^{-\mathrm{\Delta Cq}}$ , where $\mathrm{\Delta Cq}=Cq_{\text{Paraprevotella}}-Cq_{\text{total bacteria}}$ . Statistical analyses were performed using the ΔCq values, whereas $2^{-\mathrm{\Delta Cq}}$ values were used for graphical presentation of relative abundance.

### 2.12 Behavioral tests

All behavioral tests were performed in a quiet environment between 12:00 and 18:00 under double-blind conditions. Previous studies have demonstrated that NTG induced hyperalgesia peaks at 2 h post injection (Marone et al., 2018). Accordingly, mechanical pain thresholds in the hind paw and periorbital region of mice were measured 2 h after each NTG administration. In addition, basal pain thresholds were determined prior to injection. Before each test, mice were acclimated on an elevated metal mesh platform for at least 30 min. Mechanical thresholds were assessed using von Frey monofilaments (0.008–4 g) with the up-down method, starting at an intermediate intensity of 0.16 g stimulus intensity was increased in the case of a negative response (O) and decreased in the case of a positive response (X). Four additional stimuli were delivered after the first positive/negative turning point, and response patterns (e.g., OOOXOXOX) were recorded. Finally, the 50% mechanical pain threshold was calculated using an online tool^1^ (Christensen et al., 2020).

### 2.13 Immunofluorescence staining

Von Frey testing was performed 2 h after the final NTG administration. Mice were transcardially perfused, and brain tissues were collected within 30 min after completion of the behavioral testing. Mouse brain tissues were fixed overnight in 4% PFA, then dehydrated in 20 and 30% sucrose solutions sequentially until the tissue sank. The brain tissue was frozen and sectioned on a cryostat microtome (Leica CM1950, Germany) to obtain 30 μm thick sections. The sections were followed by incubation with rabbit anti-c-Fos (1:500, Cell Signaling Technology, Cat#2250), rat anti-NeuN (1:500, Oasis Biofarm, Cat#OB-PRT013) and mouse anti-CGRP (1:400, Santa Cruz Biotechnology, Cat#sc-57053) in 1 × TBS++ (TBS containing 5% donkey serum and 0.3% Triton X-100) overnight at 4 °C. Subsequently, the sections were incubated with secondary antibodies at room temperature for 2 h. All secondary antibodies were diluted at a ratio of 1:500, including donkey anti-rabbit Cy3 (Jackson ImmunoResearch Labs, Cat#711-165-152), donkey anti-mouse Cy2 (Jackson ImmunoResearch Labs, Cat#715-225-150) and donkey anti-rat Cy5 (Jackson ImmunoResearch Labs, Cat#712-175-153). The slides were mounted with medium containing 120 mg/mL polyvinyl alcohol and 31.25 mg/mL anti-fluorescence quencher 1,2-dianilinoethane. Images were acquired with a confocal microscope (Leica SP8, Germany).

### 2.14 Statistical analysis

All statistical analyses were performed using GraphPad Prism (v9.5) and R (v4.4.2). Data are presented as mean ± standard error of the mean (SEM). A *p*-value less than 0.05 was considered statistically significant. Data normality was assessed using the Shapiro–Wilk test, and the corresponding W statistics and *p* values are provided in Supplementary Table 5. For normally distributed data, comparisons among independent groups were performed using ordinary one-way ANOVA followed by Tukey’s multiple-comparisons test. Non-parametric data were analyzed using the Kruskal–Wallis test followed by Dunn’s multiple-comparisons test. Mechanical thresholds measured repeatedly over time were analyzed using two-way repeated-measures ANOVA with Geisser–Greenhouse correction followed by Šídák’s multiple-comparisons test. Mendelian randomization analysis was described in the preceding section.

## 3 Results

### 3.1 Causal associations of gut microbiota on migraine/MA/MO

A full list of the genetic instruments used for MR can be found in Supplementary Table 6. Both the primary MR results and the results after outlier removal are provided in the Supplementary Tables 7, 8. The post-outlier estimates are presented in Figure 2. Our MR analysis identified that eight gut microbiome features were causally linked to migraine and its subtypes (Bonferroni correction *p* < 3.88 × 10^−5^, 0.05/430/3; 430 is the number of gut microbiota taxa with at least one SNP and 3 represents the number of migraine types examined). For overall migraine, the abundance of *Lachnospiraceae* (odds ratio [OR] = 1.08, 95% confidence interval [CI]: 1.04–1.12, *p* = 2.58 × 10^−5^) was positively associated with the disease, while the prevalence of *Paraprevotella* (OR = 0.95, 95% CI: 0.94–0.97, *p* = 2.69 × 10^−6^) showed a negative association. For MO, higher prevalence of *Lachnospiraceae TestASV_14* (OR = 0.87, 95% CI: 0.83–0.92, *p* = 2.36 × 10^−6^), *Holdemanella OTU99_54* (OR = 0.91, 95% CI: 0.88–0.93, *p* = 4.48 × 10^−10^), *Prevotella OTU99_32* (OR = 0.92, 95% CI: 0.88–0.95, *p* = 1.68 × 10^−5^) and *Sutterella OTU97_6* (OR = 0.94, 95% CI: 0.92–0.96, *p* = 1.07 × 10^−10^) were all associated with lower risk. In contrast, higher abundance of *Prevotella OTU97_31* conferred an increased risk (OR = 1.04, 95% CI: 1.03–1.05, *p* = 1.13 × 10^−18^). For MA, an increased abundance of *Parasutterella OTU97_15* was associated with an increased risk (OR = 1.09, 95% CI: 1.05–1.14, *p* = 9.11 × 10^−6^). The scatter plots of the eight significant results are shown in Supplementary Figure 1.

**Figure 2:**
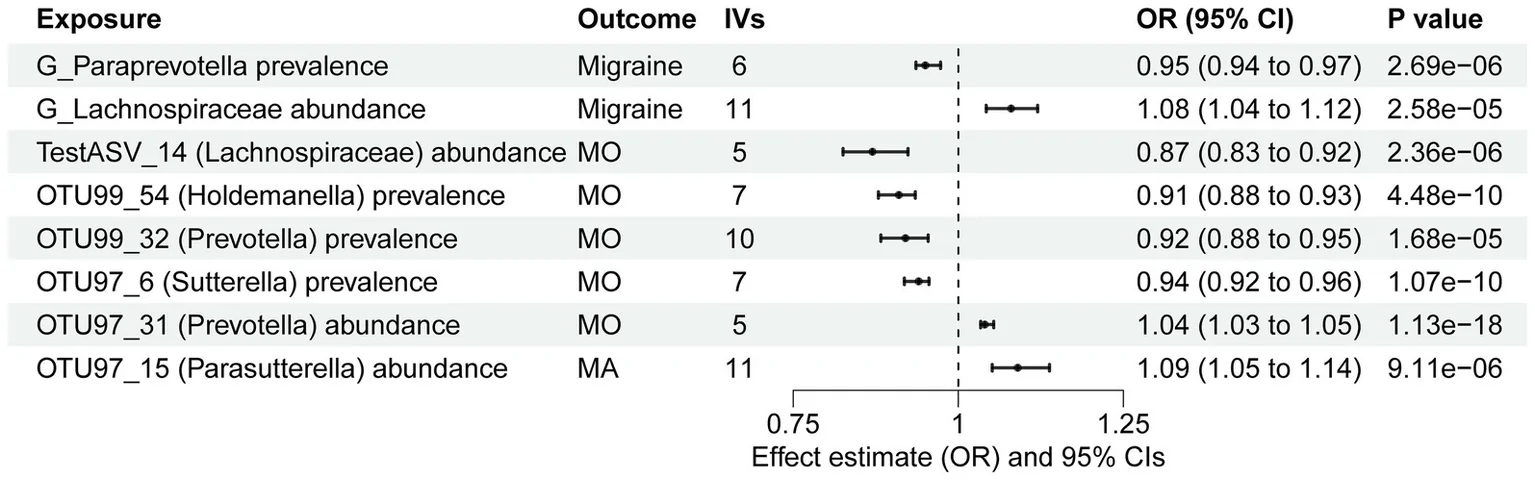
Causal effects of gut microbiome on migraine, MA, MO. Forest plot showing the significant causalities estimated using IVW or Wald ratio method. *p* < 3.88 × 10^−5^ after Bonferroni correction was considered significant. The causal estimates are expressed as OR values with 95% CI values. The width of the lines extending from the midpoint represent the 95% CI.

### 3.2 Causal effects of brain structure on migraine/MA/MO

The causal links between 2,347 brain structure phenotypes and three migraine types were assessed. Full results are provided in Supplementary Tables 9–11. IVW-MR analyses identified 30 significant associations that passed Bonferroni correction (Bonferroni correction *p* < 7.10 × 10^−6^, 0.05/2347/3; 2,347 is the number of brain MRI phenotypes with at least one SNP and 3 represents the distinct migraine types). The MR results for the top five MRI phenotypes with the largest absolute effect sizes on the three migraine types are shown in Figures 3A–C, with corresponding brain region schematics displayed in Figure 3D.

**Figure 3:**
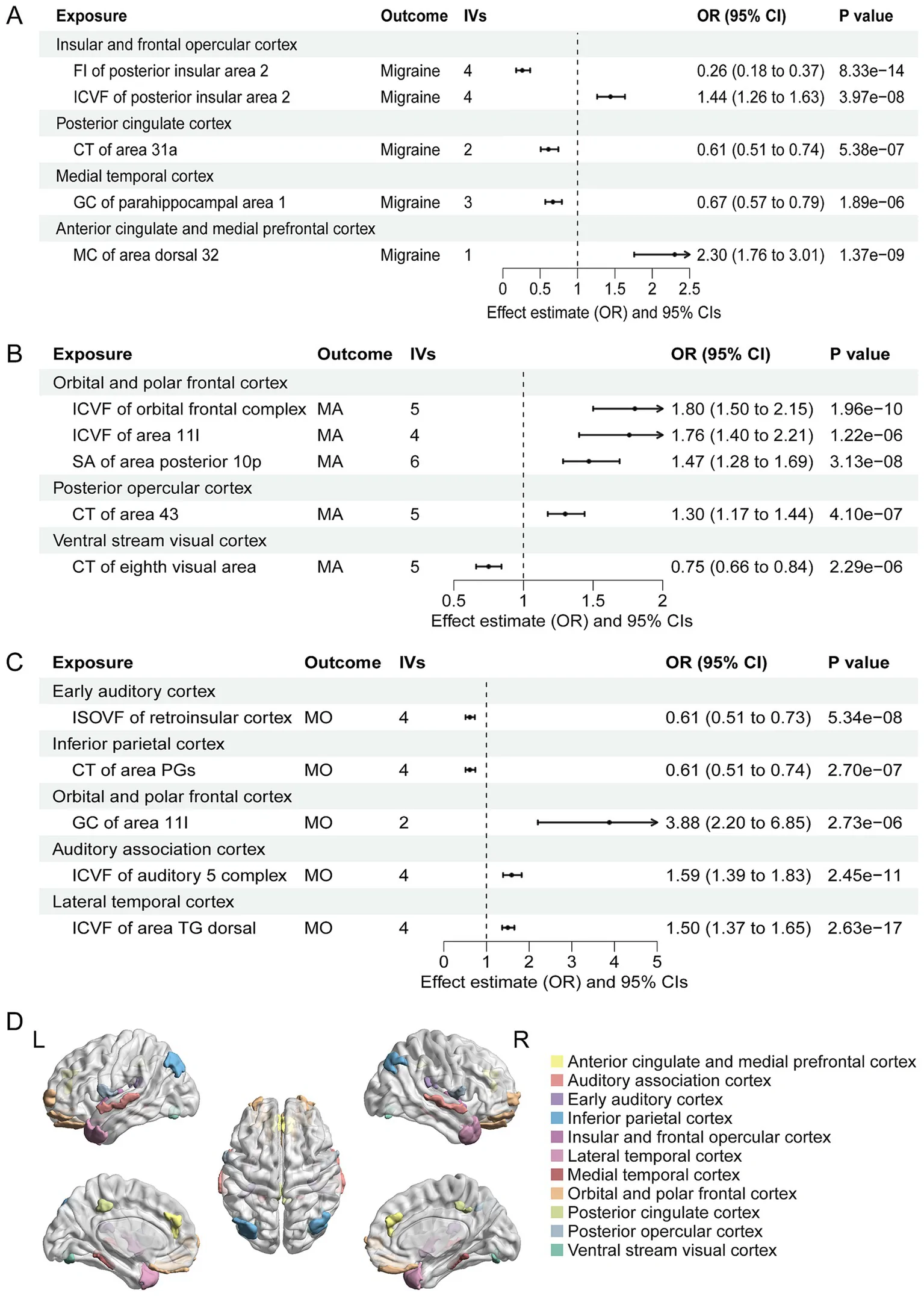
The top five causal effects of brain MRI phenotypes on migraine **(A)**, MA **(B)** and MO **(C)**. Forest plot showing the significant causalities estimated using IVW or Wald ratio method. *p* < 7.10 × 10^−6^ after Bonferroni correction was considered significant. The causal estimates are expressed as OR values with 95% CI values. Arrows indicate that the maximum interval on the x axis is extended. The width of the lines extending from the midpoint represent the 95% CI. **(D)** Schematic diagram of the brain regions in lateral, medial, and dorsal views.

For migraine, we identified 12 significant causal relationships involving multiple cortical regions. The scatter plots of the 12 results are shown in Supplementary Figure 2. Our findings demonstrated that variations in MC of the anterior cingulate and medial prefrontal cortex exerted the strongest effect on the risk of migraine (OR = 2.30, 95% CI: 1.76–3.01, *p* = 1.37 × 10^−9^). Additionally, genetically predicted CT of the ventral stream visual cortex showed a highly significant positive association with migraine risk (OR = 1.30, 95% CI: 1.24–1.37, *p* = 2.33 × 10^−25^). This association may be linked to the visual disturbances frequently experienced by migraine patients. Furthermore, multiple structure categories within the insular cortex and frontal opercular cortex were causally linked to migraine. For example, the ICVF of posterior insular area 2 was linked to a higher risk (OR = 1.44, 95% CI: 1.26–1.63, *p* = 3.97 × 10^−8^). In contrast, the FI of posterior insular area 2 in insular and frontal opercular cortex was associated with a reduced risk of migraine (OR = 0.26, 95% CI: 0.18–0.37, *p* = 8.33 × 10^−14^).

For MA, we identified five macrostructural and two microstructural phenotypes that had significant causal effects. The scatter plots of the seven results are shown in Supplementary Figure 3. It is noteworthy that three cortical phenotypes in the orbital and polar frontal cortex were associated with an increased risk. Specifically, the ICVF of orbital frontal complex showed a higher risk (OR = 1.80, 95% CI: 1.50–2.15, *p* = 1.96 × 10^−10^), as was the ICVF of area 11 L (OR = 1.76, 95% CI: 1.40–2.21, *p* = 1.22 × 10^−6^). Additionally, the SA of area posterior 10p was associated with increased risk (OR = 1.47, 95% CI: 1.28–1.69, *p* = 3.13 × 10^−8^).

For MO, 11 significant causal relationships involving multiple cortical regions were identified. Among these, auditory association cortex and inferior parietal cortex were associated with the highest number of phenotypes. Of particular note, our findings indicated that variations in GC of the orbital and polar frontal cortex showed the largest effect on the risk of developing MO (OR = 3.88, 95% CI: 2.20–6.85, *p* = 2.73 × 10^−6^). The scatter plots of the 11 results are shown in Supplementary Figure 4.

### 3.3 Mediating effect of brain structure between gut microbiota and migraine/MA/MO

Among the associations between eight migraine-related gut microbial traits and 30 neuroimaging phenotypes, three survived Bonferroni correction (*p* < 2.08 × 10^−4^). *Holdemanella OTU99_54* was positively associated with cortical thickness of posterior cingulate cortex area 31a (β = 0.05, 95% CI: 0.03–0.07, *p* = 1.02 × 10^−6^), and *Parasutterella OTU97_15* was positively associated with ICVF of the orbital frontal complex (β = 0.07, 95% CI: 0.04–0.11, *p* = 6.89 × 10^−5^). *Paraprevotella* was negatively associated with posterior insular cortex ICVF (β = −0.02, 95% CI: −0.04 to −0.01, *p* = 1.84 × 10^−4^) (Figure 4A; Supplementary Tables 12–14; Supplementary Figure 5).

**Figure 4:**
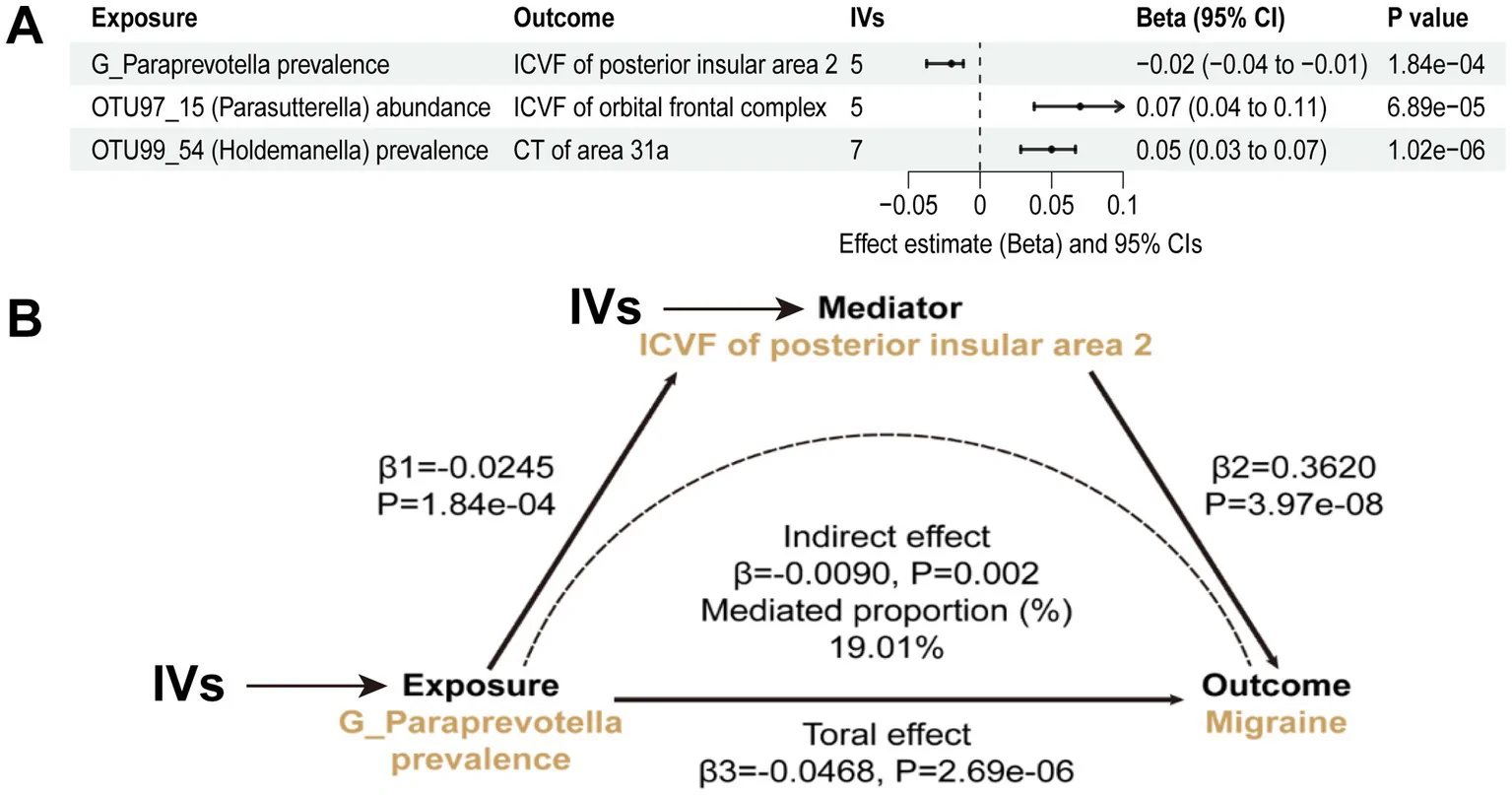
Mediation effects of the gut microbiota on migraine via microstructural alterations in the insular and frontal opercular cortex. **(A)** Causal effects of specific gut microbial taxa on brain MRI phenotypes. Forest plot showing the significant causal relationships estimated using IVW method. *p* < 2.08 × 10^−4^ after Bonferroni correction was considered significant. **(B)** Significant results from the mediation analysis.

Mediation analysis suggested that posterior insular cortex ICVF partially mediated the association between genetically predicted *Paraprevotella* abundance and migraine, with a mediated proportion of 19.01% (95% CI: 4.62–33.28%, *p* = 0.0019) (Figure 4B; Supplementary Table 15). In MVMR, the association between *Paraprevotella* and migraine remained significant after adjustment for ICVF (β = −0.273, 95% CI: −0.467 to −0.080, *p* = 0.020), whereas the adjusted effect of ICVF was not significant (β = −0.043, 95% CI: −0.089 to 0.003, *p* = 0.096) (Supplementary Table 16). These findings suggest that the association between *Paraprevotella* and migraine was not fully explained by ICVF.

Reverse MR showed no significant associations of migraine with posterior insular cortex ICVF or *Paraprevotella*. The association between posterior insular cortex ICVF and *Paraprevotella* was inconsistent across MR methods. Overall, no consistent evidence of reverse causality was observed (Supplementary Table 17).

### 3.4 Sensitivity and pleiotropy analyses

Sensitivity analyses were further performed to assess the robustness of the significant associations. Heterogeneity and potential influential variants were observed in several associations. After removal of the identified outlier SNPs (Supplementary Table 18), the MR and sensitivity analyses were repeated. Heterogeneity was markedly reduced for most associations. However, significant heterogeneity remained for cortical thickness of posterior cingulate cortex Area 31a and migraine, possibly due to the markedly different effect estimates of the two available instruments (Cochran’s Q test, *p* < 0.05). No significant evidence of horizontal pleiotropy was detected in the post-outlier analyses. The post-outlier MR estimates are presented in the main figures, and the corresponding results before and after outlier removal are provided in Supplementary Tables 6–14.

MRlap analyses were performed to account for potential sample overlap. The corrected estimates were directionally consistent with the observed estimates for the significant associations between brain structure and migraine. Complete MRlap results are provided in Supplementary Table 19. Steiger directionality analyses supported the hypothesized direction of all three associations involved in the mediation pathway (Supplementary Table 20). We did not observe significant asymmetry in funnel plots (Supplementary Figures 6–10).

### 3.5 Probiotics supplementation alleviated migraine-like behaviors in the NTG-induced mouse model of migraine

Based on the MR findings, higher genetically predicted *Paraprevotella* abundance was associated with lower migraine risk, with the ICVF of pIC identified as a potential mediator. To further explore the preventive potential of microbiome modulation *in vivo*, we employed prophylactic supplementation with compound probiotics in the mouse. In this study, 8-week-old male C57BL/6 J mice were randomly assigned to control (Vehicle-Saline), model (Vehicle-NTG) and intervention (Probiotic-NTG) groups. The intervention group received daily intragastric probiotics suspension for 21 consecutive days, while the control and model groups were given equal volumes of ddH_2_O. After the final gavage, mice were intraperitoneally injected with NTG every other day while control animals received saline instead. Basal responses were measured prior to each injection, and mechanical pain thresholds in the paw and periorbital region were assessed using von Frey filaments 2 h after NTG administration (Figure 5A). Baseline pain thresholds were similar across all groups (*p* > 0.05). Repeated NTG injection significantly decreased periorbital and hind paw pain thresholds. Compared with the model group, 21-day probiotics pretreatment effectively attenuated the NTG-induced reduction in mechanical pain threshold (Figures 5B–E). In addition, probiotics supplementation did not significantly alter either basal or post-injection periorbital or hind-paw mechanical thresholds in saline-treated mice (Supplementary Figure 11), suggesting that its effect was not attributable to a generalized alteration in baseline mechanical sensitivity. Together, prophylactic probiotics supplementation relieves NTG-induced mechanical hyperalgesia and confers protective effects against migraine.

**Figure 5:**
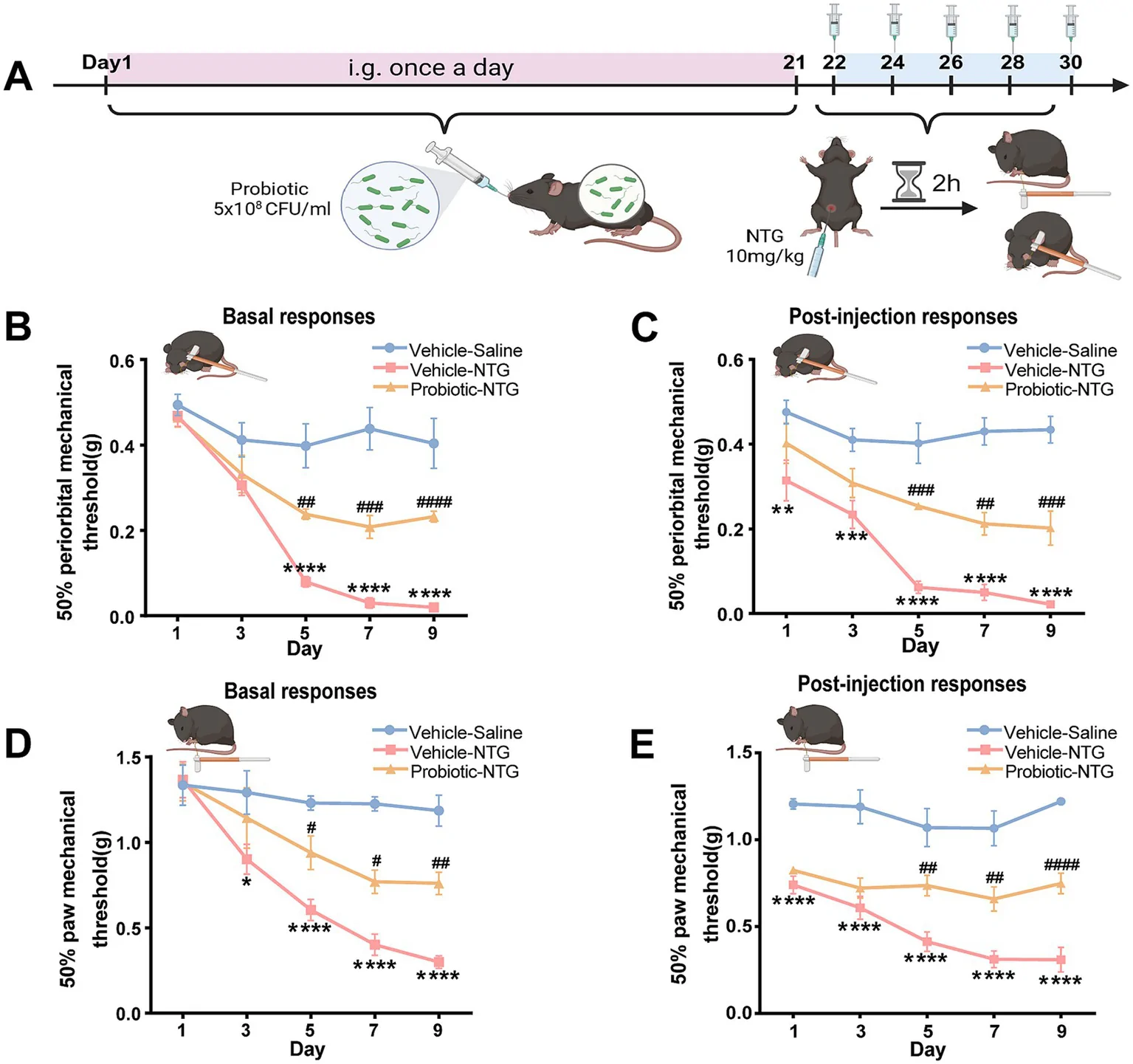
Probiotics supplementation alleviated hyperalgesia in mouse model of migraine. **(A)** Schematic diagram of experimental procedure and behavioral tests. (Created with BioRender.com). **(B–E)** Periorbital and hind-paw mechanical thresholds were assessed using von Frey filaments before each injection (basal responses) and 2 h after NTG or saline administration (post-injection responses). Statistical analysis was performed using two-way repeated-measures ANOVA with the Geisser–Greenhouse correction, followed by Šídák’s multiple-comparisons test. Group × time interaction: **(B)** *F*(8,48) = 6.412, *p* < 0.0001; **(C)** *F*(8,48) = 3.687, *p* = 0.0020; **(D)** *F*(8,48) = 4.552, *p* = 0.0004; **(E)** *F*(8,48) = 2.482, *p* = 0.0243. *n* = 5 mice per group.*p < 0.05, **p < 0.01, ***p < 0.001, ****p < 0.0001 vs. Vehicle-Saline group; #*p* < 0.05, ##*p* < 0.01, ###*p* < 0.001, ####*p* < 0.0001 vs. Vehicle-NTG group.

### 3.6 Composite probiotics pretreatment increased the abundance of *Paraprevotella* in mouse model of migraine

ACE and Chao1 indices (Figures 6A,B) demonstrated that gut microbial richness was reduced in the Vehicle-NTG model group compared with the control group (*p* < 0.05), while probiotics treatment significantly restored richness (*p* < 0.01). Meanwhile, β-diversity analysis revealed distinct clusters of the gut microbiota structure among the three groups (PERMANOVA, *p* = 0.003, Figure 6C). These findings suggest that compound probiotics intervention ameliorates gut microbiota dysbiosis in migraine model mice. We further analyzed the microbiota abundance at phylum level (Figure 6D). Given the MR findings, we then focused on the relative abundance of the identified migraine-related taxa. *Paraprevotella* abundance was significantly reduced in the Vehicle-NTG group compared with the Vehicle-Saline group and increased following probiotics supplementation (Figure 6E). Given the relatively low read counts of *Paraprevotella* (Supplementary Table 21), targeted qPCR was further performed to support the sequencing-derived abundance pattern. qPCR showed a marked decrease in the Vehicle-NTG group compared with the Vehicle-Saline group (*p* < 0.0001) and a significant increase after probiotics treatment (*p* < 0.05) (Figure 6G). Pairwise LEfSe analysis between the Vehicle-NTG and Probiotic-NTG groups further identified *Paraprevotella* as enriched following probiotics supplementation (Figure 6H). By contrast, no significant intergroup differences were observed for *Lachnospiraceae* (Figure 6F).

**Figure 6:**
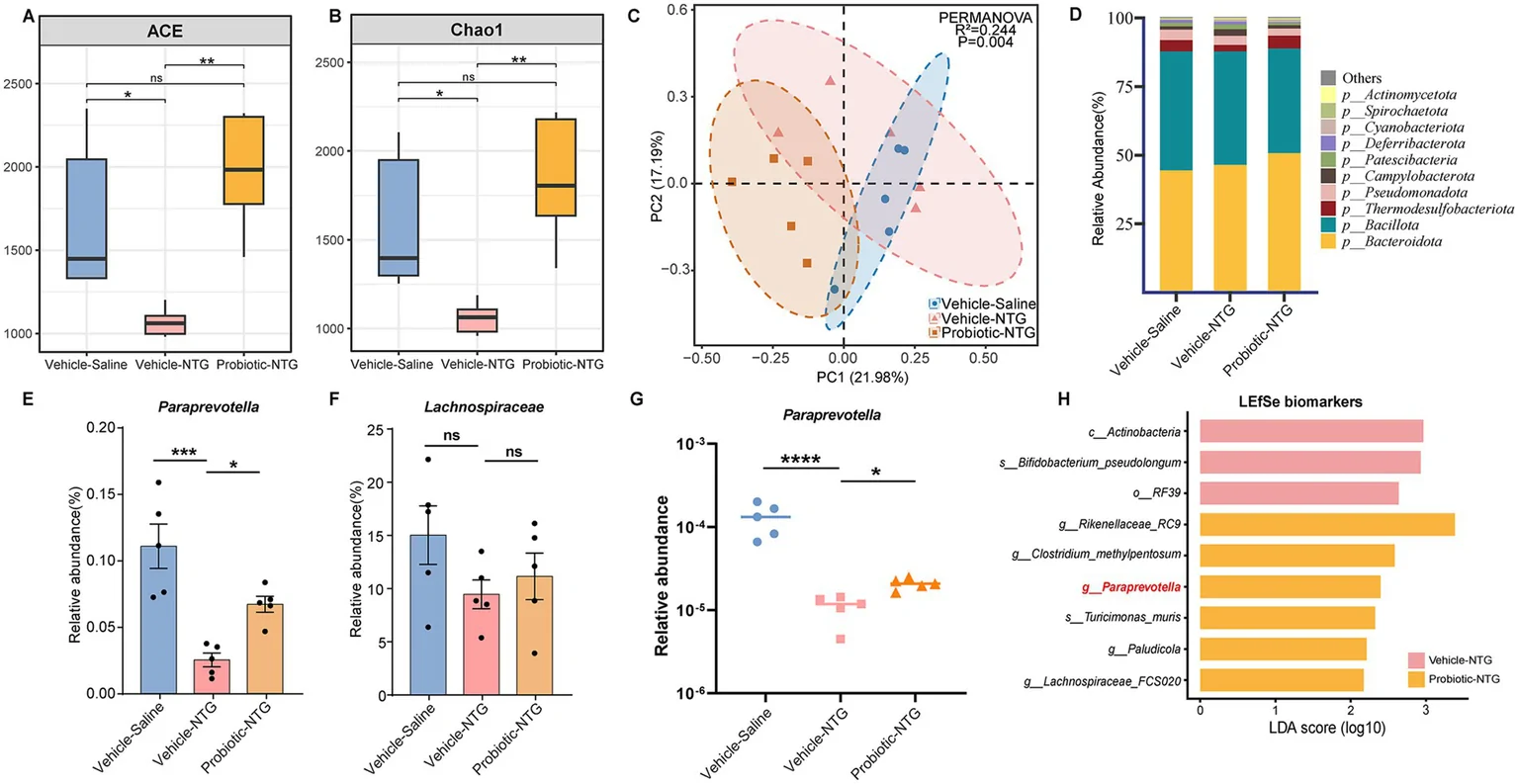
Composite probiotics pretreatment alters the gut microbiota composition in mousemodel of migraine. **(A,B)** α diversity of gut microbiota assessed by ACE and Chao1, Kruskal–Wallis test with Dunn’s multiple-comparisons test. **(C)** PCoA of β diversity using Bray-Curtisdistances. PERMDISP showed no difference in multivariate dispersion among groups (*F*(2,12) = 0.130, *p* = 0.877). **(D)** Relative abundance of the top 10 bacterial phyla. **(E,F)** Relative abundances of *Paraprevotella* **(E)** and *Lachnospiraceae* **(F)** determined by 16S rRNA gene sequencing, ordinary one-way ANOVA with Tukey’s *post hoc* test. **(G)** Relative abundance of *Paraprevotella* validated by targeted qPCR, ordinary one-way ANOVA followed by Tukey’s multiple-comparisons test. **(H)** LDA scores of biomarker taxa determined by LEfSe analysis (*p* < 0.05, LDA score ≥ 2.0). *n* = 5 mice per group. *p < 0.05, **p < 0.01, ***p < 0.001, ****p < 0.0001, ns, not significant.

### 3.7 Probiotics supplementation attenuated NTG-induced neuronal activation in the pIC region of mice

MR analysis suggested that ICVF in the posterior insular cortex (pIC) may partially mediate the association between genetically predicted *Paraprevotella* abundance and migraine risk. Since ICVF is a NODDI-derived microstructural index related to neurite density, and previous studies have demonstrated that NODDI microstructural indices correlate with brain functional connectivity (Deligianni et al., 2016; Colon-Perez et al., 2019), we further explored whether neuronal activity in the pIC was altered in the NTG-induced migraine-like model. We detected neuronal activation in the pIC using c-Fos and NeuN immunostaining. Vehicle-NTG mice displayed markedly enhanced c-Fos^+^/NeuN^+^ activation compared with controls (*p* < 0.0001). Probiotics pretreatment significantly mitigated this NTG-induced neuronal hyperactivation (*p* = 0.014) (Figures 7A–C).

**Figure 7:**
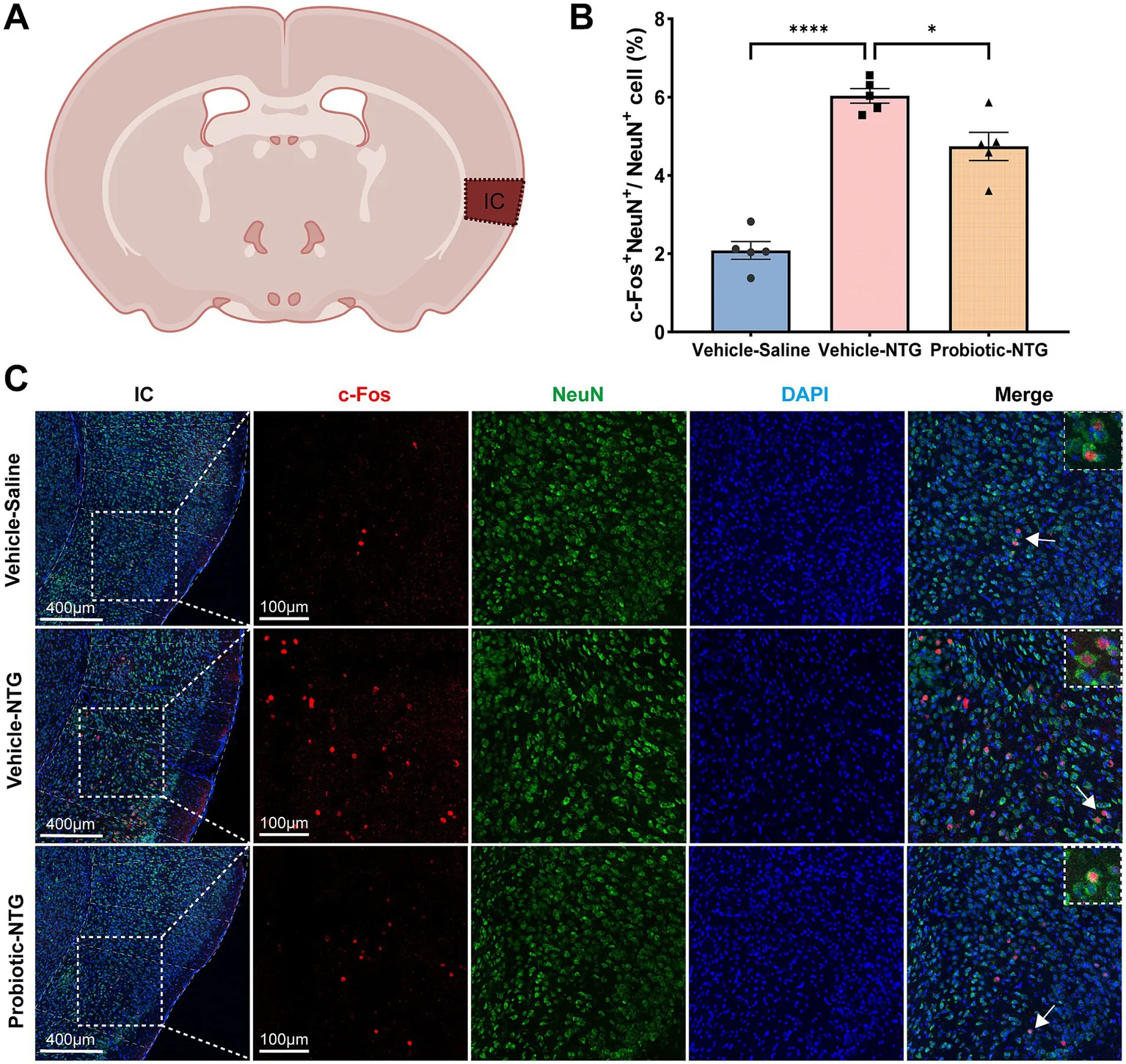
Probiotics supplementation attenuates neuronal activation in the pIC. **(A)** Schematic illustration showing anatomical location of the pIC. **(B)** The percentage of c-Fos^+^/NeuN^+^ cells in the pIC across the three groups. **(C)** Representative images showing immunofluorescence staining of c-Fos (red), NeuN (green) and DAPI (blue) in the pIC. Statistical analysis was performed using ordinary one-way ANOVA followed by Tukey’s multiple-comparisons test. *n* = 5 mice per group. *p < 0.05, ****p < 0.0001.

### 3.8 Preventive intervention with probiotics inhibits NTG-induced central sensitization in Sp5c of mice

CGRP is widely acknowledged as a migraine mediator and plays a critical role in central sensitization within the Sp5c. To further clarify the preventive effect of compound probiotics on migraine, we performed immunofluorescence staining for CGRP in the Sp5c. Compared with the control group, the mean immunofluorescence intensity of CGRP and the expression level of c-Fos were significantly upregulated in the Sp5c region of mice in the Vehicle-NTG group (*p* < 0.0001). In contrast, compared with the model group, probiotics pretreatment significantly decreased the number of c-Fos positive neurons (*p* = 0.013) and reduced CGRP immunofluorescence intensity (*p* = 0.002) (Figures 8A–E). Collectively, probiotics pretreatment attenuated NTG-induced migraine-like behaviors and was accompanied by reduced CGRP immunoreactivity and neuronal activation in the Sp5C.

**Figure 8:**
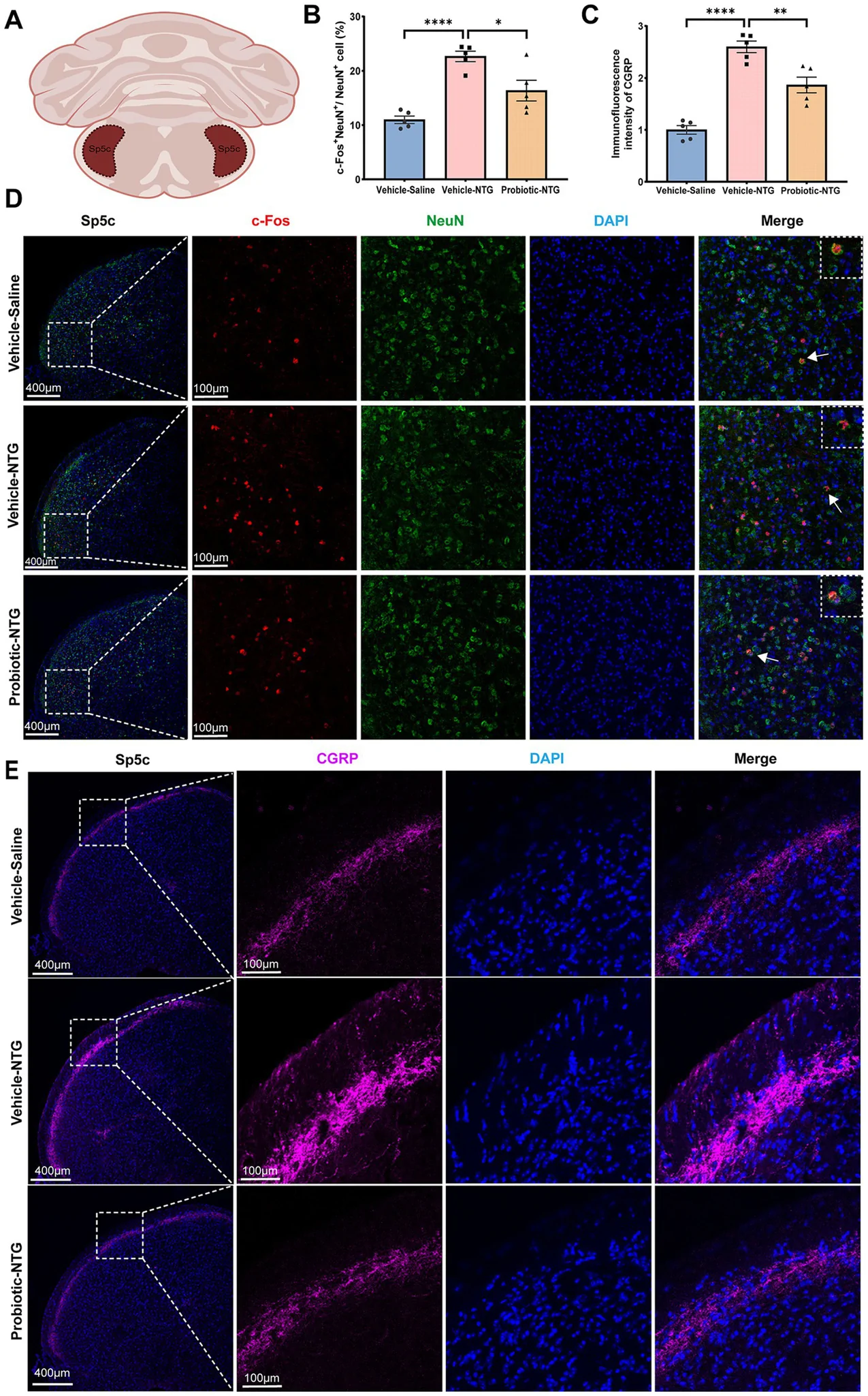
Probiotics supplementation attenuates neuronal activation in Sp5c. **(A)** Schematic illustration showing anatomical location of the Sp5c. **(B)** The percentage of c-Fos^+^/NeuN^+^ cells in the Sp5c across three groups. **(C)** Quantitative analysis of CGRP immunofluorescence intensity across three groups. **(D)** Representative images showing immunofluorescence staining of c-Fos (red), NeuN (green) and DAPI (blue). **(E)** Representative images showing immunofluorescence staining of CGRP (magenta) and DAPI (blue). Statistical analysis was performed using ordinary one-way ANOVA followed by Tukey’s multiple-comparisons test. *n* = 5 mice per group. *p < 0.05, **p < 0.01, ****p < 0.0001.

### 3.9 Probiotics pretreatment attenuates NTG-evoked neuronal activation in PVT

As a midline thalamic nucleus, the PVT is involved in modulating diverse pain states (Chang et al., 2019; Liang et al., 2020). We further examined the expression of c-Fos in the PVT. Quantitative analysis demonstrated a remarkable elevation in the ratio of c-Fos^+^ neurons among total NeuN^+^ neurons in the NTG-induced model group (*p* < 0.0001), indicating robust aberrant activation of PVT neurons following repeated NTG exposure. Notably, probiotics pretreatment significantly attenuated neuronal activation in the PVT (*p* = 0.009) (Figures 9A–C).

**Figure 9:**
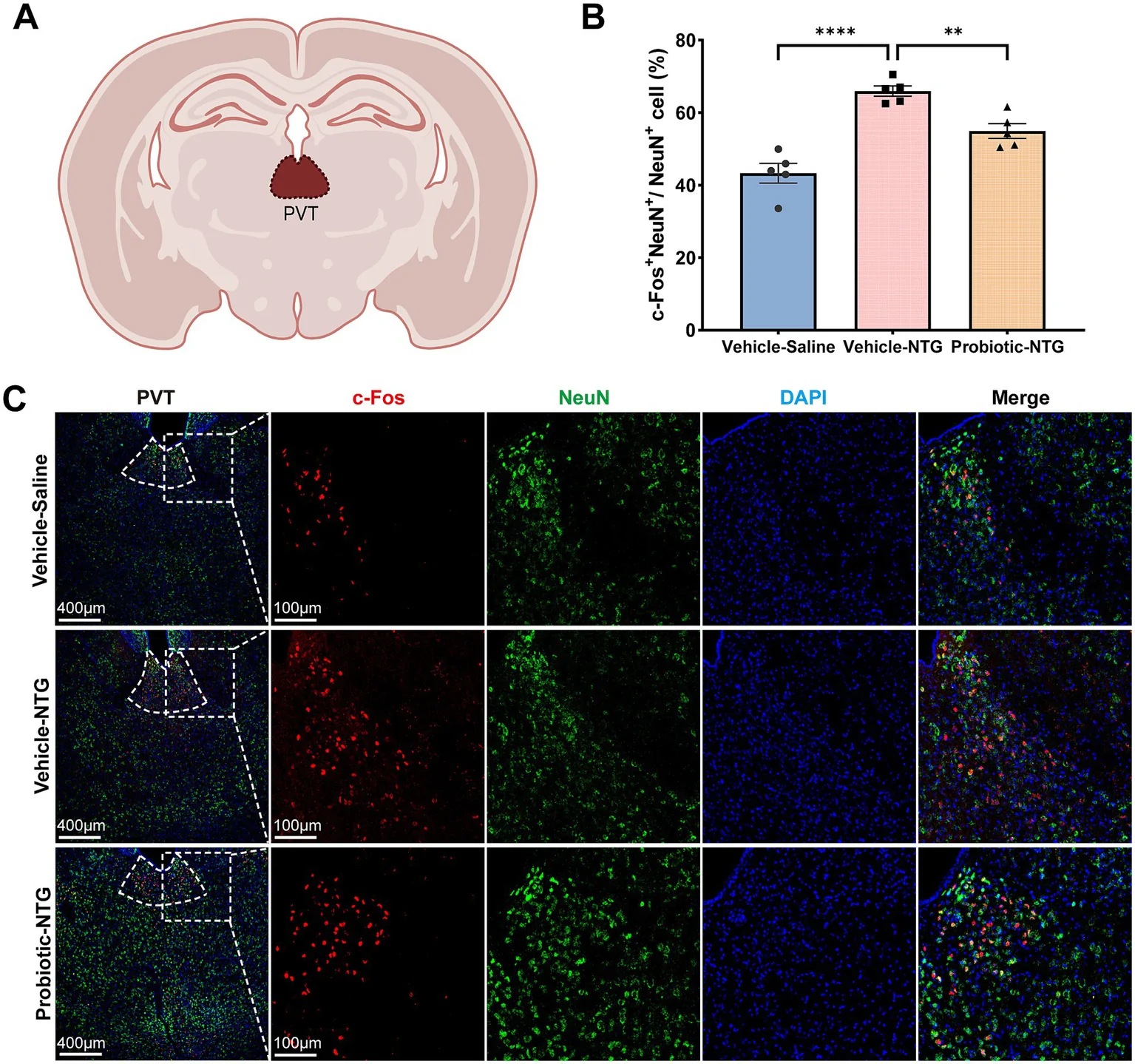
Probiotics supplementation attenuates neuronal activation in PVT. **(A)** Schematic illustration showing anatomical location of the PVT. **(B)** The percentage of c-Fos^+^/NeuN^+^ cells in PVT across three groups. **(C)** Representative images showing immunofluorescence staining of c-Fos (red), NeuN (green) and DAPI (blue). Statistical analysis was performed using ordinary one-way ANOVA followed by Tukey’s multiple-comparisons test. *n* = 5 mice per group. **p < 0.01, ****p < 0.0001.

### 3.10 Probiotics pretreatment dampens neuronal responses to NTG in mPFC

As a key region of the default mode network that mediates emotion, cognition, self-referential processing and pain modulation, the mPFC plays a critical role in the initiation and progression of migraine. The NTG-induced migraine model showed prominent c-Fos overactivation in the mPFC (*p* = 0.0001), and probiotics could attenuate such neuronal activation in migraine mice (*p* = 0.038) (Figures 10A–C).

**Figure 10:**
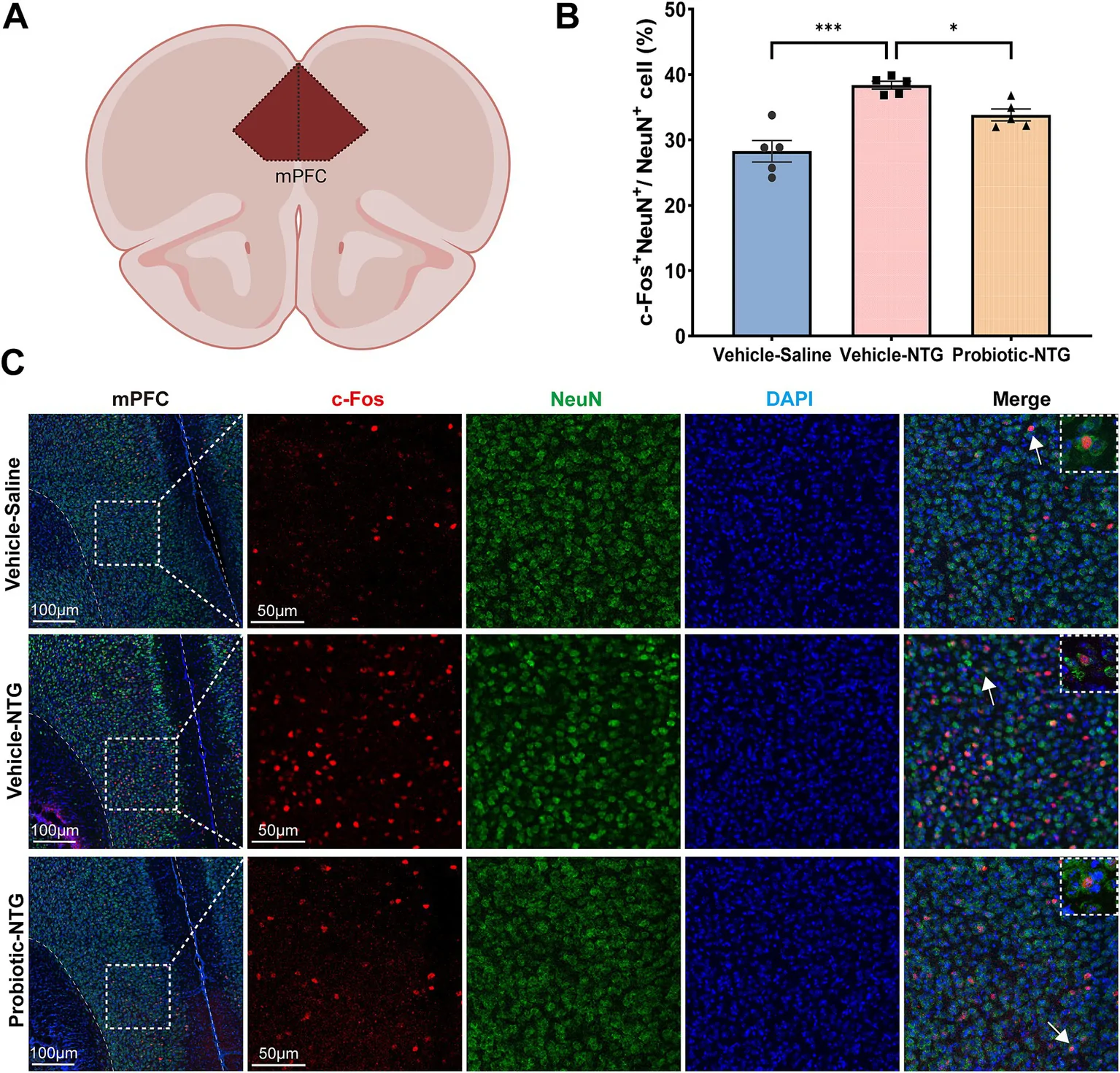
Probiotics supplementation attenuates neuronal activation in mPFC. **(A)** Schematic illustration showing anatomical location of the mPFC. **(B)** The percentage of c-Fos^+^/NeuN^+^ cells in mPFC across three groups. **(C)** Representative images showing immunofluorescence staining of c-Fos (red), NeuN (green) and DAPI (blue). Statistical analysis was performed using ordinary one-way ANOVA followed by Tukey’s multiple-comparisons test. *n* = 5 mice per group. *p < 0.05, ***p < 0.001.

## 4 Discussion

MGBA dysfunction is emerging as a pathogenic mechanism in neurological disorders (Aburto and Cryan, 2024; Ohara and Hsiao, 2025). In particular, migraine patients suffer from intestinal dysbiosis, which may influence the onset and progression of the disease (Arzani et al., 2020; Mugo et al., 2025). Collectively, this study provides complementary genetic and experimental evidence supporting a potential role of the gut microbiome in migraine. MR analyses identified several microbial taxa associated with migraine risk and suggested ICVF in the pIC as a potential mediator of the association between genetically predicted *Paraprevotella* abundance and migraine. Consistent with these findings, prophylactic compound probiotics supplementation attenuated NTG-induced mechanical hypersensitivity and neuronal hyperactivation in mice, accompanied by the maintenance of *Paraprevotella* abundance. Together, these findings provide an integrated genetic and experimental perspective on the MGBA in migraine and support microbiome-targeted modulation as a promising strategy for migraine prevention.

Emerging evidence has increasingly linked migraine to gastrointestinal symptoms (Aurora et al., 2021). The intestinal epithelium detects signals from the gut microbiota and transmits these signals to the central nervous system, thereby mediating the perception of pain (Meerschaert and Chiu, 2025). Accumulating evidence further supports an association between alterations in the gut microbiota and migraine (Arzani et al., 2020; Socała et al., 2021). In the present study, our MR analysis identified eight microbial features that passed Bonferroni correction, including *Lachnospiraceae, Paraprevotella, Lachnospiraceae TestASV_14, Holdemanella OTU99_54, Prevotella OTU99_32, Sutterella OTU97_6, Prevotella OTU97_31, and Parasutterella OTU97_15*. A clinical study reported a lower abundance of *Paraprevotella* in patients with migraine and IBS compared with patients with IBS alone, which is directionally consistent with the protective association observed for genetically predicted higher *Paraprevotella* abundance in our study (Liu et al., 2022a). Similarly, a previous bidirectional MR study identified *Lachnospiraceae* as a potential risk factor for migraine, which is consistent with the positive association observed for the feature in our study (Qu et al., 2024). Notably, *Lachnospiraceae TestASV_14* showed an inverse association with MO in our analysis, in contrast to the positive association observed for the broader *Lachnospiraceae* feature. This discrepancy may reflect substantial heterogeneity within the *Lachnospiraceae* family, with distinct taxa or sequence-defined features potentially exerting different effects on migraine susceptibility. In our mouse experiments, NTG exposure was associated with a reduction in the relative abundance of *Paraprevotella*, whereas prophylactic compound probiotics supplementation partially restored its abundance. Furthermore, a double-blind randomized controlled trial demonstrated that 10 weeks of supplementation with 14 probiotics strains significantly reduced both the frequency and severity of migraine attacks (Martami et al., 2019), supporting the broader potential of microbiome-targeted interventions for migraine prevention.

Although the link between gut microbiome perturbations and migraine has been extensively documented in observational studies (Chen et al., 2019; Kappéter et al., 2023; Yong et al., 2023), the underlying mechanistic basis of this relationship remains incompletely understood. The MGBA may influence migraine through several metabolic and immune pathways, including tryptophan metabolism and inflammatory signaling (Cryan et al., 2019; Morais et al., 2021). Altered tryptophan metabolism may influence migraine through serotonergic signaling. Gut microorganisms can modulate serotonin biosynthesis, and changes in gut microbiota and circulating serotonin levels have been reported in patients with migraine (Yano et al., 2015; Liu et al., 2024). The clinical efficacy of the selective 5-HT1F receptor agonist lasmiditan further supports the relevance of serotonergic signaling to migraine (Goadsby et al., 2019). In parallel, gut dysbiosis may promote inflammatory signaling involved in migraine-related nociception. Sustained TNF-α upregulation has been associated with prolonged migraine-like pain, whereas TNF-α blockade attenuated this effect (Tang et al., 2020). In patients with migraine, synbiotic supplementation also reduced attack frequency, serum hs-CRP, and zonulin levels (Ghavami et al., 2021).

Accumulating evidence indicates that gut microbiome modulates brain structure (Tillisch et al., 2017; Lu et al., 2018). Combining these findings, we speculate brain structure may play a crucial mediating role in the gut microbiota’s influence on migraine. Step-by-step screening and subsequent MR mediation analysis demonstrated that the causal effect of *Paraprevotella* on migraine was partially (19.01%) mediated by the ICVF of the pIC. As a potent trypsin degrader, a reduction in *Paraprevotella* abundance may lead to the accumulation of trypsin in the gut (Li et al., 2022). Excess trypsin could overstimulate intestinal protease-activated receptor 2 (PAR2), which is likely to disrupt intestinal barrier integrity and allow trypsin leakage into systemic circulation (Bianchimano et al., 2023; Lakemeyer et al., 2025). Once reaching the dura mater, circulating trypsin may further activate PAR2 on trigeminal nociceptive fibers. Such PAR2 hyperactivation might subsequently elicit acute headache-like behavioral responses and render individuals more vulnerable to headache provocation by NO donors (Mason et al., 2023). Previous studies have reported that monoclonal antibodies targeting PAR2 exert certain therapeutic effects in mouse models of migraine. Additionally, studies have demonstrated that trypsin increases c-Fos expression in the brain through PAR2 signaling (Alsrhani et al., 2021). These inflammatory signals and/or associated immune mediators are then thought to propagate to the central nervous system, potentially via compromised barrier function, neuroinflammation, or neuronal sensitization, ultimately driving the cortical changes implicated in migraine pathogenesis. In addition, *Paraprevotella* is involved in the metabolism of short-chain fatty acids (SCFAs). A marked decrease in its abundance has been associated with reduced levels of SCFAs in both the intestine and the brain. This decline in SCFAs may further elevate pro-inflammatory cytokines and oxidative stress in brain tissue, potentially leading to structural brain alterations (Gao et al., 2021; Du et al., 2024).

Previous studies have demonstrated that migraine pathogenesis represents a multilevel neural network regulatory process, involving brainstem primary nociceptive receptive nuclei, thalamic signal relay centers, and higher-order cognitive-emotional cortices in concert (Ferrari et al., 2022). Neuroanatomically, pain signals transmit via an ordered serial pathway. Specifically, the spinal trigeminal nucleus caudalis (Sp5c) integrates meningeal trigeminal nociception in the medulla. This signal is then relayed through the thalamus to cortical areas (Ashina, 2020; Ferrari et al., 2022). The pIC produces subjective pain perception as the core integrative center (Frot et al., 2014; Kadakia et al., 2024), whereas the mPFC modulates pain via descending and emotional regulatory pathways (Ong et al., 2019; Lv et al., 2024). By examining c-Fos expression across these pain-related regions, our findings suggest that probiotics pretreatment attenuated neuronal hyperactivation within the migraine-related nociceptive network. These findings provide a strong rationale for targeting the gut–brain axis as a potential therapeutic strategy in clinical practice, offering a novel avenue for migraine prevention and treatment. However, the potential influence of behavioral testing on c-Fos protein activation cannot be ignored. Previous studies have shown that c-Fos protein expression following acute nociceptive or mechanical stimulation typically reaches robust or maximal levels approximately 1–2 h after stimulation (Gao and Ji, 2009; Liu et al., 2022b; Ke et al., 2024). Therefore, brain tissues were collected within 30 min after behavioral testing to minimize this potential confounding effect.

This study has several strengths and limitations. A major strength is the use of comprehensive GWAS data from 8,956 German individuals, which enabled refined taxonomic resolution of gut microbial composition. In addition, the preventive effects of the compound probiotic formulation observed in this study support further investigation of microbiome-targeted strategies for migraine prevention. However, several limitations should be considered. First, the exclusive use of European ancestry GWAS datasets may limit the generalizability of our findings to other populations. Second, only male mice were used to minimize variability related to estrogen and estrous-cycle fluctuations, which limits the assessment of sex-specific effects given the female predominance of migraine. Third, probiotic efficacy may vary according to strain, dose, treatment duration, and formulation. Differences in baseline gut microbiota, migraine phenotype, and the multifactorial pathophysiology of migraine may further contribute to variability in treatment response. Therefore, the probiotic findings should be interpreted in the context of the specific regimen used in this study. Finally, the specific contribution of *Paraprevotella* remains unclear, and ICVF-related brain microstructure was not directly evaluated in the animal model. Future studies should include sex-balanced models and further evaluate defined probiotic strains and doses, *Paraprevotella*, and brain microstructure.

## 5 Conclusion

In summary, this study highlights the microbiota–gut–brain axis as a potentially important framework for understanding migraine pathophysiology. The findings support a link between gut microbial variation, brain microstructural changes, and migraine susceptibility, and further suggest that modulation of the gut microbiota may represent a promising avenue for migraine prevention. Future studies are needed to clarify the underlying biological mechanisms and to evaluate the translational potential of microbiome-targeted interventions.

## Data Availability

The 16S rRNA sequencing data generated in this study have been deposited in the NCBI Sequence Read Archive (SRA) under BioProject accession number PRJNA1542051 (https://www.ncbi.nlm.nih.gov/sra/PRJNA1542051).The original contributions presented in the study are included in the article/Supplementary material, further inquiries can be directed to the corresponding author.

## Footnotes

1
https://bioapps.shinyapps.io/von_frey_app/
