# Supplementary material for: Preventive effects of compound probiotics supplementation on migraine: Mendelian randomization analysis and experimental evidence
Source: Front Microbiol. 2026 Oct 21;17:1939532. doi: 10.3389/fmicb.2026.1939532 (PMC13732465; doi:10.3389/fmicb.2026.1939532)
Supplement: Supplementary file 1 [file Supplementary_file_1.docx]

**Supplementary Figure 1.** Scatter plots of genetic associations between gut microbiota and migraine, MA, and MO.

**Supplementary Figure 2.** Scatter plots of genetic associations between brain MRI phenotypes and migraine.

**Supplementary Figure 3.** Scatter plots of genetic associations between brain MRI phenotypes and MA.

**Supplementary Figure 4.** Scatter plots of genetic associations between brain MRI phenotypes and MO.

**Supplementary Figure 5.** Scatter plots of genetic associations between gut microbiota and brain MRI phenotypes.

**Supplementary Figure 6.** Funnel plots for gut microbiota on migraine, MA and MO.

**Supplementary Figure 7.** Funnel plots for brain MRI phenotypes on migraine.

**Supplementary Figure 8.** Funnel plots for brain MRI phenotypes on MA.

**Supplementary Figure 9.** Funnel plots for brain MRI phenotypes on MO.

**Supplementary Figure 10.** Funnel plots for gut microbiota on brain MRI phenotypes.

**Supplementary Figure 11.** Probiotic supplementation does not significantly alter mechanical sensitivity under saline conditions. Periorbital (A, B) and hind-paw (C, D) mechanical withdrawal thresholds were assessed in Vehicle- Saline and Probiotic -Saline mice under basal and post-injection condition. Statistical analysis was performed using two-way repeated-measures ANOVA with the Geisser–Greenhouse correction, followed by Šídák’s multiple-comparisons test. Group × time interaction: (A) *F*(4,32) = 0.1995, *P* = 0.9368; (B) *F*(4,32) = 0.1960, *P* = 0.9387; (C) *F*(4,32) = 0.07661, *P* = 0.9889; (D) *F*(4,32) = 0.6338, *P* = 0.6421. No significant between-group differences were detected at individual time points. n = 5 mice per group.
